# Fully automated segmentation of clinical target volume in cervical cancer from magnetic resonance imaging with convolutional neural network

**DOI:** 10.1002/acm2.13725

**Published:** 2022-07-27

**Authors:** Fatemeh Zabihollahy, Akila N. Viswanathan, Ehud J. Schmidt, Junghoon Lee

**Affiliations:** ^1^ Department of Radiation Oncology and Molecular Radiation Sciences Johns Hopkins University School of Medicine Baltimore Maryland USA; ^2^ Division of Cardiology, Department of Medicine Johns Hopkins University School of Medicine Baltimore Maryland USA

**Keywords:** cervical cancer, clinical target volume, deep learning segmentation, magnetic resonance imaging, radiotherapy

## Abstract

**Purpose:**

Contouring clinical target volume (CTV) from medical images is an essential step for radiotherapy (RT) planning. Magnetic resonance imaging (MRI) is used as a standard imaging modality for CTV segmentation in cervical cancer due to its superior soft‐tissue contrast. However, the delineation of CTV is challenging as CTV contains microscopic extensions that are not clearly visible even in MR images, resulting in significant contour variability among radiation oncologists depending on their knowledge and experience. In this study, we propose a fully automated deep learning–based method to segment CTV from MR images.

**Methods:**

Our method begins with the bladder segmentation, from which the CTV position is estimated in the axial view. The superior–inferior CTV span is then detected using an Attention U‐Net. A CTV‐specific region of interest (ROI) is determined, and three‐dimensional (3‐D) blocks are extracted from the ROI volume. Finally, a CTV segmentation map is computed using a 3‐D U‐Net from the extracted 3‐D blocks.

**Results:**

We developed and evaluated our method using 213 MRI scans obtained from 125 patients (183 for training, 30 for test). Our method achieved (mean ± SD) Dice similarity coefficient of 0.85 ± 0.03 and the 95th percentile Hausdorff distance of 3.70 ± 0.35 mm on test cases, outperforming other state‐of‐the‐art methods significantly (*p*‐value < 0.05). Our method also produces an uncertainty map along with the CTV segmentation by employing the Monte Carlo dropout technique to draw physician's attention to the regions with high uncertainty, where careful review and manual correction may be needed.

**Conclusions:**

Experimental results show that the developed method is accurate, fast, and reproducible for contouring CTV from MRI, demonstrating its potential to assist radiation oncologists in alleviating the burden of tedious contouring for RT planning in cervical cancer.

## 1 INTRODUCTION

**FIGURE 1:**
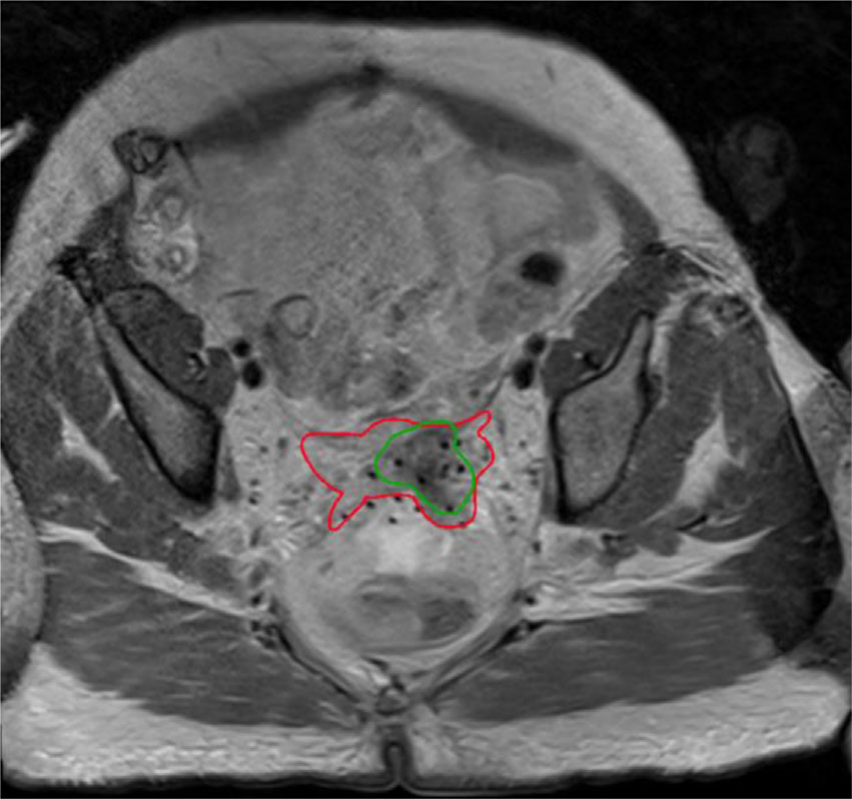
An example of clinical target volume (CTV) (red) and gross tumor volume (GTV) (green) segmentation on an magnetic resonance imaging (MRI) taken at the time of brachytherapy

External beam radiation therapy (EBRT) followed by brachytherapy (BT) is a standard treatment option for patients with cervical cancer. Research demonstrates that BT that involves placing radiation sources inside the target tumor region through temporary catheters improves survival rates by 30%–40% over EBRT alone.^1^ Computed tomography (CT) is routinely used to generate volumetric information of the body for both EBRT planning and applicator localization in BT. However, in cervical cancer, T2‐weighted (T2W) magnetic resonance imaging (MRI) is considered the gold standard for defining the extent of tumor involvement due to its high soft‐tissue contrast.^2^, ^3^ Once the MR image is obtained, accurate delineation of the target tumor and nearby normal organs at risk (OAR) is required for radiotherapy (RT) planning. Although automatic OAR segmentation has been widely studied, showing promising results, accurate tumor segmentation has been less frequently explored. In a typical RT setting, the gross tumor volume (GTV) that is the visible tumor on MR images is contoured by the attending radiation oncologist. Physicians use their knowledge of the disease to expand the GTV and generate a contour for clinical target volume (CTV), which includes the microscopic extensions of tumor not clearly visible even in MRI^4^ (Figure 1). As such, CTV delineation relies on a mixture of predefined and judgment‐based margins, which makes this process particularly a difficult task, leading to high intra‐ and interobserver variability. Eminowicz et al.^5^ reported the existence of significant interobserver variation in cervical cancer CTV delineation and the necessity of ongoing effort to ensure interobserver consistency through education, guidelines, and multicenter collaboration. Although different guidelines were published defining CTV for RT planning,^6^ there are differences between guidelines that preclude adopting one method as a gold standard for CTV identification. Even using a particular guideline leads to discrepancies among CTV contours generated by different physicians. Dimopoulos et al.^7^ performed an interobserver comparison of CTV contouring when using the GYN GEC‐ESTRO guideline for MRI‐assisted cervical cancer BT and observed significant differences between the mean volumes of the outlined pairs of contours, as drawn by different observers from different institutes for the intermediate‐risk CTV. Therefore, segmenting CTV, manually or automatically, is very challenging, and an accurate, consistent, and efficient delineation of CTV from MRI is essential for optimal RT planning.

Ghose et al.^8^ provided a review of conventional computer‐aided methods developed for adaptive treatment planning and RT planning for cervical cancer using CT and MR images. Most prior CTV segmentation methods were intended for adaptive replanning, wherein contours are transferred from the planning images to treatment day images or replanning images using a deformable image registration (DIR).^9^, ^10^, ^11^, ^12^ The DIR‐based approach may be used for segmenting CTV for a different patient with a new planning image, but it cannot robustly deal with large anatomical variations that exist in CTV, thus yielding poor segmentation results.^12^

Recently deep learning (DL)‐based methods have been used to automatically segment CTV from CT and MR images.^13^, ^14^, ^15^, ^16^, ^17^ These studies proposed effective convolutional neural network (CNN) architectures for target region segmentation by combining U‐Net with an elegant feature extraction architecture. To our knowledge, only one study focused on cervical cancer CTV segmentation from female pelvic CT images; Liu et al.^18^ proposed a method in which the whole U‐Net encoder part was replaced with residual^19^ block and dense block (DB)^20^ combined into a dual path architecture to simultaneously segment CTV and OAR. DL‐based CTV segmentation methods have been proposed for other target sites. Balagopal et al.^14^ proposed a coarse‐to‐fine segmentation method in which a two‐dimensional (2‐D) U‐Net is first used to localize CTV and its nearby OAR, including the bladder, rectum, femoral heads, and penile bulb in a prostate CT scan. The auto‐segmented bladder and rectum masks filled with the corresponding CT numbers are used as an extra input channel for a three‐dimensional (3‐D) U‐Net to segment CTV. The suggested method achieved an average Dice similarity coefficient (DSC) of 0.87 ± 0.05 on a test dataset of 50 patients. Elguindi et al.^21^ employed DeepLabV3+ to segment OAR and CTV from male pelvic T2W MR images and achieved DSC of 0.83 ± 0.06 for prostate and seminal vesicles (considered CTV) on 10 test images. Jin et al.^16^ integrated RT CT and positron emission tomography (PET) images into a two‐stream chained deep fusion framework to segment esophageal cancer GTV. As the CTV encompasses the GTV and the involved lymph nodes that are somewhat distant from the OAR, the CTV segmentation problem was formulated as a deep contextual appearance‐based problem using encoded spatial distances of these anatomical structures. Fourfold cross‐validation on 148 esophageal cancer patients yielded a DSC of 0.83 ± 0.05. Liu et al.^15^ used a U‐Net with a residual convolutional layer as the building block to segment the CTV of breast cancer for RT and reported the mean DSC of 0.90 ± 0.02 on 21 test cases. Xu et al.^17^ segmented CTV for stomach cancer by a stochastic width deep neural network in which shortcut connections between the encoder and decoder were employed randomly. The proposed algorithm was evaluated using 150 cases using a fivefold cross‐validation method and gave a mean DSC of 0.74. Men et al.^13^ proposed a deep dilated convolutional neural network (DDCNN) to segment CTV in the planning CT for rectal cancer. DDCNN employs a multiple‐scale convolutional architecture to extract multiple‐scale context features in the early layers, which contain the original information on fine texture and boundaries. In addition, it enlarged the receptive fields of dilated convolutions at the end of networks to capture complementary context features. DDCNN achieved a DSC of 0.88 on 60 test cases.

In this work, we propose a fully automated DL‐based workflow to accurately and efficiently contour the high‐risk CTV (HR‐CTV) from MRI that is needed for BT that follows an EBRT. Although one study focused on CT‐based CTV segmentation for cervical cancer RT,^18^ no study has been performed to segment CTV from MRI for cervical cancer RT planning. Our proposed pipeline consists of three steps: bladder localization, CTV span detection, and final CTV segmentation. We first locate the bladder using a 3‐D U‐Net to roughly estimate the location of the CTV with respect to the bladder position. The input MR images are cropped around the estimated CTV location in the axial direction. A 2‐D Attention U‐Net is used for every cropped axial slice image to detect the CTV span along the superior–inferior (SI) direction, from which a 3‐D CTV‐specific region of interest (ROI) is computed. The original MRI volume is cropped using the ROI and input to the CTV segmentation network. For the final CTV segmentation, we use a 3‐D block‐based U‐Net (B2 U‐Net) that uses 3‐D patch block within the ROI and slides through the SI direction. 3‐D CNN‐based methods have demonstrated better performance in medical image segmentation than 2‐D CNNs as they allow for a better exploration of the inter‐slice context information.^22^ However, implementing and properly training complex 3‐D network requires a large amount of annotated data, which is not readily available in many cases. Furthermore, CTV size varies from patient to patient, which makes a single 3‐D network with a fixed input volume size ineffective. 3‐D B2 U‐Net addresses these issues by benefiting from 3‐D processing, reduced computational complexity, and flexibly accommodates varying CTV shape and size. This approach also increases the number of training samples by extracting smaller 3‐D volumes from the ROI, which is crucial for properly training the network.

DL‐based CTV segmentation results typically have uncertainties due to the absence of clear CTV boundaries in the image. Providing uncertainty information of the model prediction helps physicians better interpret and improve the automatic segmentation result especially for regions with high uncertainty. To estimate uncertainty, we followed the approach in Gal et al.^23^ which used a regular dropout to estimate posterior distribution of the Gaussian process. Normally, dropout is used as a regularization technique^24^ during training, where different sets of neurons are randomly dropped out in every training iteration; hence different model architectures are trained each time. In the testing phase, the dropout is switched off and the outcome is an averaging ensemble of many different neural networks. Turning the dropout layer on in the testing phase provides Monte Carlo (MC) samples from the space of all available models that can be used for Bayesian inference (posterior distribution) approximation. This novel framework, referred to as MC dropout (MCDO), is a useful tool for representing uncertainty in medical image processing. In this study, we compute an uncertainty map along with CTV segmentation to offer secondary information of the segmentation quality so that the physician can carefully review segmented regions with high uncertainty and revise them as needed.

The novelty of the proposed approach is threefold:

- We developed an anatomy‐guided pipeline to segment CTV fully automatically in cervical cancer RT from MR images. 3‐D CNN‐based modules were embedded in the proposed method, to capture inter‐slice contextual information while addressing computational costs associated with the 3‐D model complexity.
- In the proposed method, CTV is segmented from a single MR imaging sequence, that is, T2W, which eliminates the need for registering multimodal images, for example, multi‐parametric MRIs, PET/CT, or CT, which is often needed in many other approaches.^16^, ^25^, ^26^ To our knowledge, this is the first attempt to segment CTV from female pelvic MR images.
- We estimate uncertainty for CTV segmentation to draw physicians’ attention to the segmentation regions with large uncertainty that may need revision.

## 2 MATERIALS AND METHODS

**FIGURE 2:**
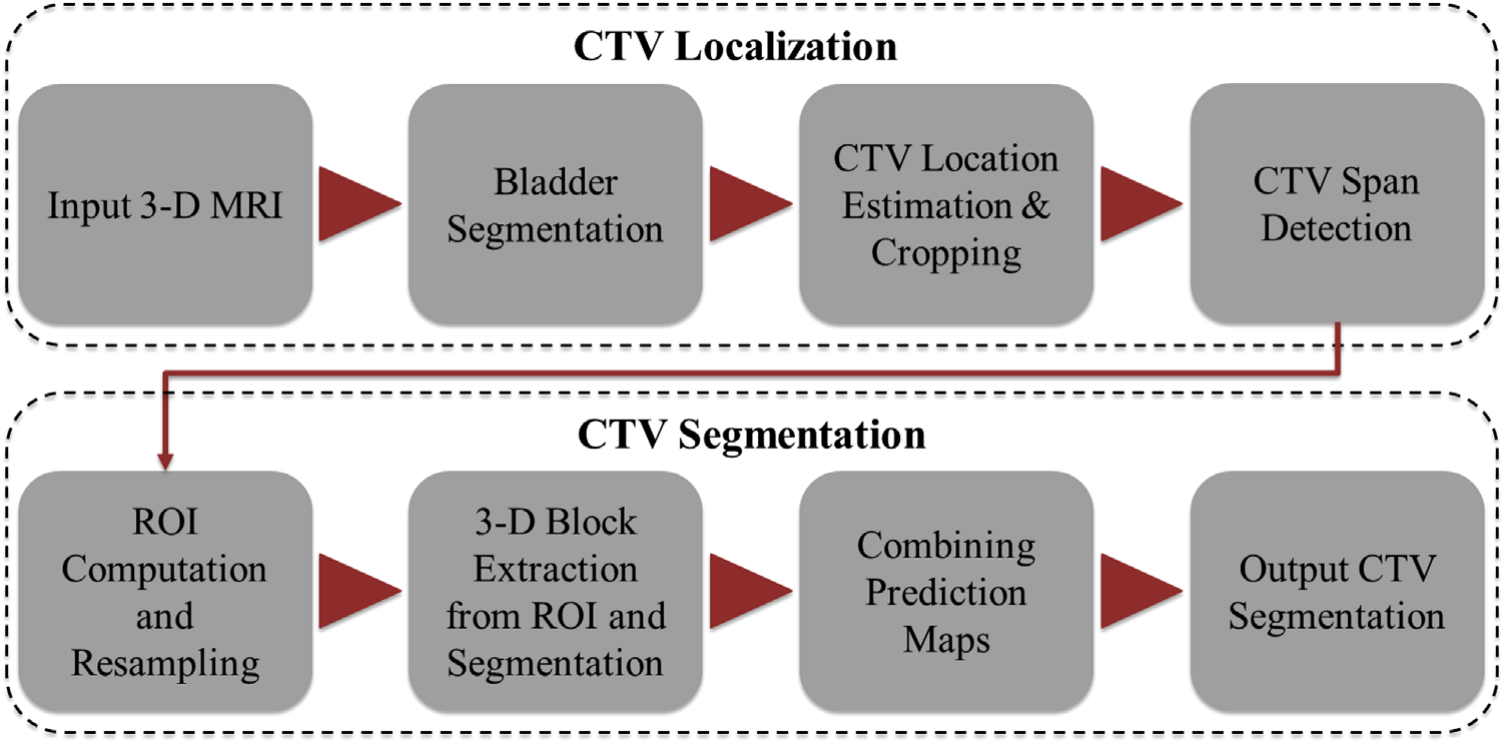
The proposed workflow for automatic clinical target volume (CTV) segmentation

Figure 2 shows an overview of our method. We employ a cascaded strategy to first localize CTV in MRI and from there segment CTV. CTV localization consists of two sequential steps. We first segment the bladder using a 3‐D U‐Net and use its positional information to estimate the CTV location. In the second step, a 2‐D Attention U‐Net is employed to identify the CTV span along the SI direction, from which the CTV‐specific ROI is computed. The original MRI volume is then cropped using the ROI. Finally, 3‐D blocks, that is, smaller 3‐D sub‐volumes, are extracted from the ROI volume from which CTV segmentation maps are computed using a 3‐D U‐Net and compiled to produce the final CTV segmentation.

### 2.1 Datasets

With the approval of the institutional review board, 213 T2W MR image sets were collected from a total of 125 patients (note that some patients had multiple MRI scans) who underwent RT at our institution. T2W MR images were obtained as part of a routine RT planning process using a Siemens Espree 1.5T scanner (Siemens Healthineers, Malvern, PA) with a 3‐D T2W SPACE (sampling perfection with application‐optimized contrasts using different flip angle evolution) sequence (TR = 2600 ms, TE = 95 ms, and slice thickness = 1.60 mm). MR images have 640 × 580 × (104–144) voxels with a voxel size of 0.5625 × 0.5625 × 1.60 mm^3^. The obtained images were manually contoured first by radiation oncology residents followed by review and correction by an attending radiation oncologist specializing in gynecological cancer for RT planning. As preprocessing, histogram equalization was performed to improve the MR image contrast, and the MR image intensities were normalized to [0, 1] to ensure that each input volume had a similar intensity distribution.

### 2.2 CTV localization

As the original MRI volume contains a large background, the entire pelvic area, that carries contextual information irrelevant to the CTV, we first extract the CTV‐specific ROI from the input MRI volume. This step improves the segmentation performance by discarding features from irrelevant backgrounds. Moreover, localization allows us to reduce the input volume size, thus reducing the computational burden. Siddique et al.^27^ summarized the benefits and applications of the cascaded network, including cascaded U‐Net for medical image segmentation. Our localization approach is a two‐step process. In the first step, the bladder is segmented by a 3‐D U‐Net, followed by the estimation of the CTV location with respect to the bladder. We observed that in our dataset, the CTV center is located on average approximately 4‐cm posterior to the center of the bladder. We therefore crop each axial image to an ROI size of 128 × 128 and pixel size of 1 × 1 mm^2^ across all axial slices, which produces large enough axial ROI images (but much smaller than the original MR images) without missing the CTV. Finally, the CTV span along the SI direction is determined by using a 2‐D Attention U‐Net, which allows us to generate a 3‐D CTV‐specific ROI volume for final segmentation.

#### 2.2.1 Bladder segmentation

The goal of the bladder segmentation is to estimate the potential CTV location relative to the bladder. This step does not require a sophisticated segmentation model to get the highest quality segmentation like in recent studies,^28^, ^29^, ^30^, ^31^ but a conventional model with reasonable performance is sufficient. We therefore cropped and resampled the original MR volume around the center with a size of 94 × 94 × 50 voxels and an enlarged voxel size of 3.0 × 3.0 × 3.2 mm^3^ and employed a standard 3‐D U‐Net^22^ that consists of contraction and expansion paths each with four layers. For the contraction path, the number of kernels is set to 16 in the first layer that doubles at the successive layers. The expansion path has a symmetrical architecture of the contraction path. In each layer, a 3 × 3 × 3 convolution, followed by batch normalization, and a rectified linear unit (ReLU) activation is performed. At the final stage, a 1 × 1 × 1 convolution followed by the sigmoid activation is applied to all feature maps to generate a probability map. The probability map is then thresholded to produce the binary label map.

#### 2.2.2 CTV location estimation

**FIGURE 3:**
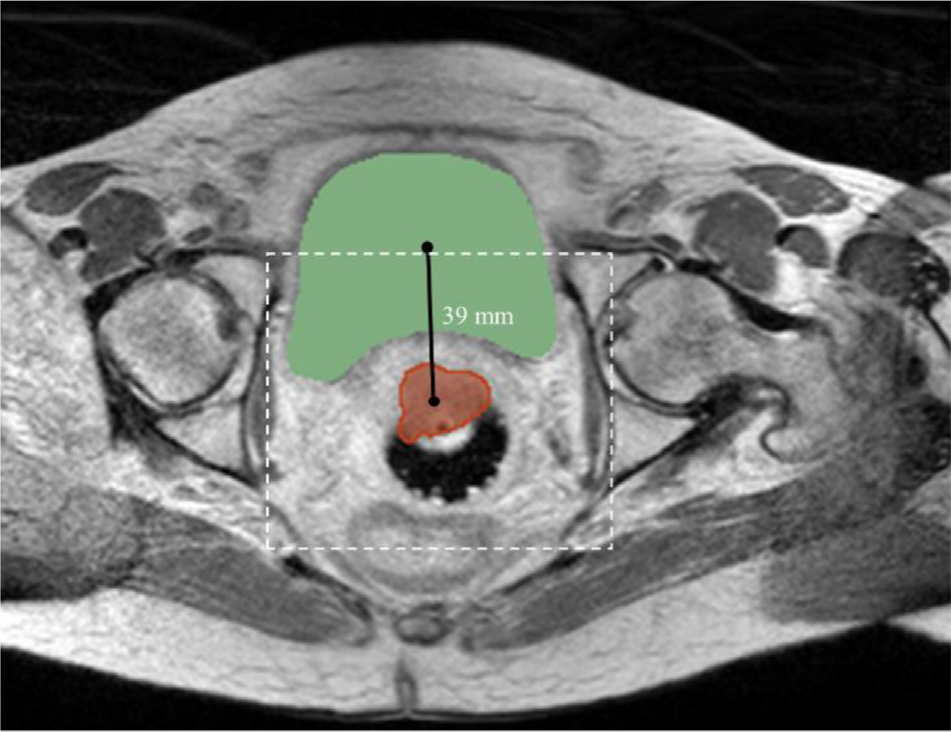
An example showing the bladder and clinical target volume (CTV) locational relationship along with the cropped region of interest (ROI) (white‐dashed window) in a typical female pelvic axial magnetic resonance imaging (MRI) in our dataset

Once the bladder is segmented, its centroid is computed from the label map and used to estimate the CTV center in the axial view as shown in Figure 3. A 128 × 128 window around the estimated CTV center is then cropped in the axial MR image (with a pixel size of 1 × 1 mm^2^). The cropped 2‐D axial MR image is fed to a 2‐D Attention U‐Net that segments CTV from the cropped 2‐D image. Once all axial images are segmented, the CTV span across the SI direction is determined from the segmentations. We used a 2‐D network as the CTV span significantly varies across SI direction patient by patient, and it is challenging to determine a proper 3‐D input volume size (especially a number of slices across the SI direction) for a 3‐D segmentation network. Our 2‐D‐based approach allows us to determine which axial slice contains CTV or not. Finally, a 3‐D CTV‐specific ROI is computed from the segmentation maps for final CTV segmentation.

We use Attention U‐Net to detect CTV span due to the benefit of adding attention gate (AG) to consistently improve the prediction performance of base architectures while preserving computational efficiency.^32^ A 2‐D U‐Net architecture used as the backbone comprises contraction and expansion paths each with five layers. The number of kernels is set to 16 in the first layer that doubles at the successive layers. In each layer, a 3 × 3 convolution, followed by batch normalization, and an ReLU activation are performed. At the final stage, a 1 × 1 convolution followed by the sigmoid activation is applied to all feature maps to generate a probability map.

Due to the location of the cervix relative to the bladder, the CTV is located posterior to the bladder. We analyzed our datasets and found that the CTV center is located 39 ± 10 mm (mean ± standard deviation [SD]) posterior to the bladder center while being laterally aligned. Therefore, we crop and resample each 2‐D axial slice image to have 128 × 128 pixels with a pixel size of 1 × 1 mm^2^ centered at this estimated CTV center and input the cropped image to the Attention U‐Net (CTV span detection network). CTV is typically shown in approximately one‐third of the SI span of our MRI volume, which may cause a class imbalance issue. Therefore, we applied random shift when extracting the 2‐D slice images crossing CTV to augment the training samples showing CTV.

The detection network typically produces some false positives that appear as sparse small regions. Morphological cleaning was applied to the output of the detection network to remove small false positives that appeared on the segmentation map.

### 2.3 CTV segmentation

CTV segmentation network, which uses a 3‐D U‐Net, takes the CTV‐specific ROI volume obtained from the CTV localization step as input to precisely segment CTV. We extract 3‐D blocks, each with a size of 128 × 128 × 15 voxels and a voxel size of 1 × 1 × 1.6 mm^3^, from ROI to reduce computational complexity while increasing the number of the training samples. As 3‐D blocks are extracted with a stride of 1, there are overlaps among adjacent blocks, leading to more than one prediction for each voxel. For final prediction, the predicted label maps are combined via union rule, where a voxel is labeled as CTV if it has been assigned to CTV at least once. We empirically chose this approach instead of majority voting approach as it gave us better segmentation performance (discussed in Section 4). We compared this strategy with conventional 2‐D/3‐D‐based network approaches where a single prediction was made for each voxel (presented in Section 3). The architecture of the 3‐D U‐Net used for CTV segmentation is similar to what is used for bladder segmentation with the difference that 32 filters are used in the first layer of the network and doubled in the subsequent layers.

### 2.4 Implementation details

DSC was used as a metric to be evaluated by all networks in the proposed pipeline for CTV segmentation during training that were trained separately. DSC is computed as 2|V_A_∩V_M_|/(|V_A_|+|V_M_|) where V_A_ and V_M_ denote automatic and manual segmentations, respectively. AdaDelta was used as the optimizer as it does not require manual tuning for the learning rate. Additionally, AdaDelta is robust to noisy gradients, differences in CNN architecture, various data modalities, and hyper‐parameter selection.^33^

A total of 213 MR images obtained from 125 patients were split into 183 (from 112 patients) for training and 30 (from 13 patients) for test. Overall, 10% of the training samples were used for validation during training. A total of 910 3‐D MR volumes, 25400 2‐D slices, and 4397 3‐D blocks extracted from ROI were used to train the bladder segmentation, CTV span detection, and CTV segmentation networks, respectively. All the networks were trained for 100 epochs on the training dataset. Validation metrics were monitored, and the best model was saved as well in conjunction with the training. For all networks, the best saved model was compared with the one saved at the end of 100 epochs, and the network with higher performance was selected for the test.

The predicted segmentation mask (defined in the ROI volume) was resampled to the original MRI volume space to produce the final segmentation. The proposed networks were implemented in Python using Keras‐TensorFlow. All networks were trained and tested on a workstation with an Intel Xeon processor (Intel, Santa Clara, CA) with 32‐GB RAM, and an NVIDIA GeForce GTX Titan X graphics processing unit (GPU) with 12‐GB memory (NVIDIA, Santa Clara, CA).

### 2.5 Uncertainty estimation

We used MCDO to capture the uncertainty of the CTV segmentation. We turned on the dropout layer during the test to produce MC samples. The number of MC samples was set to 100 to measure the uncertainty within a reasonable time while generating enough samples to produce confident results. Consequently, there were 100 different probability values for each voxel in the MRI. The mean value of 100 predictions was calculated to produce the uncertainty map, which illustrates the level of uncertainty for each voxel inside the CTV.

### 2.6 Evaluation metrics

The performance of the proposed method was quantitatively evaluated using multiple metrics in comparison to the attending radiation oncologist's manual segmentation. We used DSC as a measure of spatial overlap between the network‐generated and the manual segmentations. Absolute volume difference (AVD) was calculated as a volume‐based metric. Additionally, Hausdorff distance (HD95) was measured by measuring the 95th percentile of the distance between the surface points of the network‐generated and manual segmentations. All metrics were computed in 3‐D for each case and the average across all cases in the test dataset was reported. A two‐sided paired *t*‐test with *α* = 0.05 was conducted to investigate the significance of the difference between segmentations computed by different methods compared to our proposed method.

## 3 RESULTS

**FIGURE 4:**
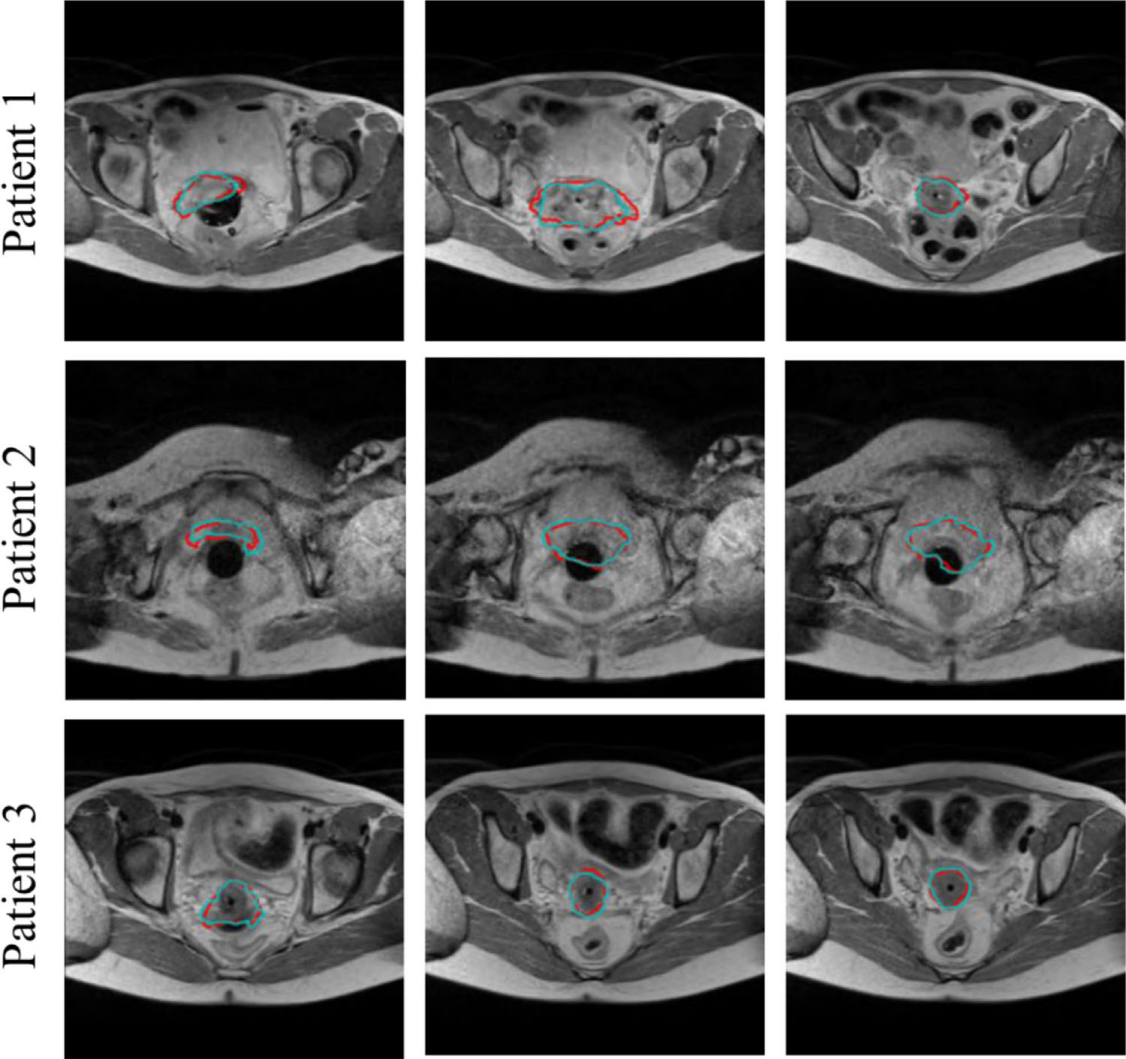
Examples of clinical target volume (CTV) segmentations from MR images using the proposed method: (red) automatic segmentation, (cyan) manual segmentation

The proposed method was evaluated on 30 test cases, and Figure 4 shows examples of test MR images from three subjects overlaid with the CTV segmentations computed by the proposed method, as compared to the expert's manual segmentations. Our method yielded DSC, AVD, and HD95 (mean ± SD) of 0.85 ± 0.03, 13.47 ± 10.01 cm^3^, and 3.70 ± 0.35 mm. Average computation time, including preprocessing, bladder segmentation, CTV location estimation, and CTV segmentation on the test cases, is 41.23 ± 7.79 s. The results demonstrate that our proposed method is capable of accurately segmenting CTV from MR images.

### 3.1 Ablation study

An ablation study was conducted to investigate the impact of each component of the proposed method. We compared the DSC values of the segmented CTVs predicted by the following networks that are similar to the proposed method with the difference being that (1) anatomy‐guided module (i.e., bladder segmentation) was removed from the pipeline, (2) CTV span detection was omitted from the CTV localization workflow, and CTV segmentation was performed using (3) 2‐D Attention U‐Net, (4) 3‐D Dense U‐Net, (5) 3‐D U‐Net, (6) 3‐D B2 Dense U‐Net, and (7) 3‐D B2 Attention U‐Net. Table 1 summarizes the methods.

**TABLE 1:** Ablation study results for clinical target volume (CTV) segmentation on three‐dimensional (3‐D) magnetic resonance (MR) images against expert manual segmentation

Method	Bladder localization	CTV span detection	CTV segmentation	DSC	*p*‐Value
1	–	2‐D Attention U‐Net	3‐D B2 U‐Net	*0.81 ± 0.08	0.0274
2	3‐D U‐Net	–	3‐D B2 U‐Net	*0.78 ± 0.05	1.81E − 08
3	3‐D U‐Net	2‐D Attention U‐Net	2‐D Attention U‐Net	*0.80 ± 0.05	7.17E − 05
4	3‐D U‐Net	2‐D Attention U‐Net	3‐D Dense U‐Net	*0.80 ± 0.10	0.0131
5	3‐D U‐Net	2‐D Attention U‐Net	3‐D U‐Net	*0.81 ± 0.08	0.0265
6	3‐D U‐Net	2‐D Attention U‐Net	3‐D B2 Dense U‐Net	*0.82 ± 0.05	0.0082
7	3‐D U‐Net	2‐D Attention U‐Net	3‐D B2 Attention U‐Net	*0.80 ± 0.06	1.25E − 04
Proposed	3‐D U‐Net	2‐D Attention U‐Net	3‐D B2 U‐Net	**0.85** ± **0.03**	–

*Note*: The asterisk denotes statistical significance using the *t*‐test performed to compare the average DSC value reported from our proposed method against those of alternatives.

Abbreviations: 2‐D, two‐dimensional; 3‐D, three‐dimensional; DSC, Dice similarity coefficient.

In the 3‐D Dense U‐Net used in our experiments, the second convolution in every layer of the 3‐D U‐Net (used for bladder segmentation) is replaced by a 3‐D DB. The DB comprises three layers where the number of feature maps in the first layer is 8 and is multiplied by three in the subsequent layers. In each layer, a 3 × 3 × 3 convolution, followed by batch normalization, and ReLU activation is performed. For methods 3–7, we used the same CTV localization step to find the ROI but used different segmentation networks. The same preprocessing of the MR images was performed for all the networks. The false positives produced by the detection model were removed similarly for all alternative methods except method 2 in which CTV span detection is removed from the proposed workflow.

Table 1 shows the results of the ablation study. Our proposed method presents the highest DSC with the smallest SD compared to the other methods, which further indicates the effectiveness and robustness of our proposed method. In the first two tests, anatomy guidance and CTV span detection were removed, respectively, from the pipeline to investigate the importance of these steps for CTV localization and successive CTV segmentation. Using bladder positional information and CTV span detection network increased the CTV segmentation accuracy by 4% and 7%, respectively. In methods 3–7, CTV segmentation was performed using different state‐of‐the‐art 2‐D, 3‐D, and 3‐D B2‐based networks to evaluate the effectiveness of the proposed method for fine CTV segmentation. The 3‐D B2 U‐Net outperformed all the other networks with statistically significant difference (two‐sided *t*‐test, *p*‐value < 0.05).

Comparing DSC obtained using 3‐D U‐Net and 3‐D B2 U‐Net demonstrates the usefulness of employing a block‐based approach for our application. Although both architectures benefit from inter‐slice information by applying 3‐D convolutional operation, the block‐based strategy allows for using higher image resolution (voxel size of 1 × 1 × 1.6 mm^3^ for 3‐D B2 CNN‐based vs. 1 × 1 × 3.2 mm^3^ for 3‐D CNN‐based) given limited computing resource, that is, GPU memory, due to its smaller input volume size (128 × 128 × 15 voxels for 3‐D B2 CNN‐based vs. 128 × 128 × 40 voxels for 3‐D CNN‐based). As we only reduce the number of 2‐D slices in the extracted blocks (compared to the whole number of slices in ROI) while keeping the axial plane the same (i.e., 128 × 128), all global features that exist in axial view are preserved. Moreover, extracting 3‐D blocks from ROI creates a large number of training samples. For 3‐D CNN‐based network training, we augmented the training images by applying random translation and rotation operations. Although this augmentation still maintains the CTV shape in the augmented training images, CTV shown in training images for 3‐D B2 CNN networks varies substantially as each block contains only a portion of CTV. We believe this helps more robust training of the 3‐D B2 CNN networks.

As we extract 3‐D blocks with a stride of one, there is overlap between adjacent blocks, yielding more than one prediction for each voxel, which we believe leads to a more confident prediction. Comparison results (Table 1) show that 3‐D B2 Dense and Attention U‐Nets yielded a poorer segmentation performance than the 3‐D B2 U‐Net. It might be due to overfitting as adding DB and AG into the 3‐D U‐Net increases the capacity of the network, and also due to the increased network complexity while the training data remains the same.

**FIGURE 5:**
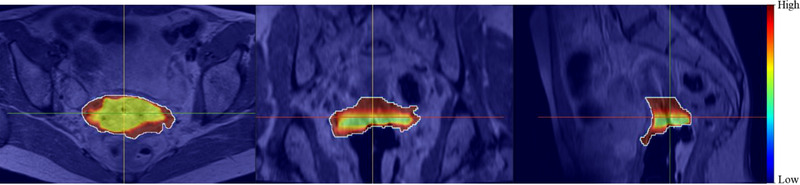
An example of an uncertainty map (blue–red heatmap) overlaid with MR image and the clinical target volume (CTV) segmentation (white contour)

An example of an uncertainty map (represented as a blue–red heatmap) overlaid with MR images and the CTV segmentation computed by our method is displayed in Figure 5. The red color is associated with the regions with large uncertainty, where the predicted segmentation may or may not agree well with the manual segmentation. For those regions, the attending physician has to decide whether to accept or revise the segmentation. The blue–yellow color in the produced uncertainty map is related to the areas, where estimated uncertainty is small, and the model‐predicted segmentation is likely to match well with the expert's manual segmentation. As expected, the uncertainty map demonstrates that the model is quite confident for the central region segmentation but less certain closer to the boundary.

## 4 DISCUSSION

We developed a cascaded CNN approach to automatically segment CTV from T2W MRI for cervical cancer RT planning, which is the first attempt of applying DL‐based techniques for this application. Previous studies show that despite following published guidelines, there are large variabilities in physicians’ manual CTV contours.^7^ The main contribution of this work is that our proposed method is fully automated without requiring any input from the user. This method along with our previously developed algorithm for automatic OAR segmentation^31^ provides an important automated tool for cervical cancer RT planning.

Due to the anatomical location of the cervix, cervical cancer CTV is surrounded by other organs such as the rectum, bladder, and femoral heads. This prior information was embedded in our pipeline to locate CTV in MRI. The results of our previous study demonstrated that the bladder, rectum, and sigmoid can be accurately segmented from MRI.^31^ As the bladder can be robustly and accurately segmented by using a DL model and the cervix is located posterior to the bladder, we picked the bladder as the guide anatomy to localize CTV. Another network was cascaded to the bladder segmentation model to detect CTV span along the SI direction. The results of the ablation study confirm that adding these two modules significantly improved CTV segmentation accuracy compared to other methods, where CTV localization is performed using only one of these modules. For CTV segmentation, a simple yet efficient 3‐D B2 U‐Net was employed that extracts 3‐D blocks from ROI and fed it to a 3‐D U‐Net for segmentation. This strategy facilitates using inter‐slice information that exists in 3‐D MRI with the highest image resolution possible when computational resource is limited. This approach allows us to increase the number of training data, that is, we extracted 4397 3‐D blocks from 213 original MR images. As we slide the 3‐D block through SI direction, slices overlap between adjacent 3‐D blocks, leading to multiple predictions for each voxel in the test stage. This likely leads to more accurate prediction compared to methods that produce one prediction per voxel. We combined those predictions and assign a voxel to CTV if the voxel has been predicted as CTV at least once. This combination rule yielded higher DSC compared to the majority voting strategy (0.85 ± 0.03 vs. 0.83 ± 0.05).

Another contribution of this study is that our method estimates segmentation uncertainty, which allows the physician to better interpret and efficiently review the automatic segmentation results to determine whether to accept or revise it, especially for regions with high uncertainty. We used the MCDO method to measure uncertainty in which the dropout layer is active in the test step. Therefore, when the test runs for multiple times, different probability values will be predicted for each voxel in MRI. The mean value of all predictions is computed to generate the uncertainty map. Case‐by‐case assessment of the results revealed that the high uncertainty estimated from our method is manifested near the CTV boundaries, where physicians are often not sure to include or exclude the region into the CTV. However, for most cases, the segmented CTV contours match well with the physician's manual segmentation, implying that no or only minor revisions may be necessary before being used for RT planning. Uncertainty evaluation adds an average of 189.83 ± 74.70 s to the time required for automatic CTV segmentation. One way to assess the quality of the estimated uncertainty is to perform multi‐observer contouring study, in which we can estimate the experts’ contour variability and compute manual segmentation uncertainty (i.e., variability) to which the network computed uncertainty map can be compared to. We anticipate that the network estimated uncertainty map would be distributed similarly as the uncertainty in the manual segmentations, which is our future research direction.

The main goal of this study was to segment HR‐CTV from female pelvic MRI that is needed for BT that follows an EBRT. MRI is considered the gold standard for CTV segmentation as it is not feasible to contour HR‐CTV on CT or cone‐beam CT due to the lack of visibility of CTV on CT scans. Even on MRI, manual delineation of CTV is a challenging task even for expert radiation oncologists as the tumor and its microscopic extension are not clearly visible, leading to variability in contouring among different delineators. There are a handful of studies proposing a DL approach to segment CTV on CT for EBRT planning, which includes much larger regions surrounded by several soft tissues/organs.^34^, ^35^, ^36^ Therefore, contouring EBRT CTV can be done on CT as it can be guided by the normal organs and the nodes that are visible in CT although their image contrast is not as good as in MRI. We have previously developed DL‐based cascaded coarse‐to‐fine segmentation models to segment male and female pelvic CT and MRI,^37^, ^38^ and these DL models can be used to segment the EBRT CTV on CT.

Previously Liu et al.^18^ proposed a 2.5‐D CNN‐based method to segment CTV from CT that is the only DL‐based method developed for cervical cancer CTV segmentation in EBRT. The authors combined GTV, cervix, vagina, parametria, uterus, and nodal CTV to generate the CTV that reported DSC of 0.86 ± 0.04 on 27 test cases. Unlike EBRT that typically treats the primary and nodal GTVs as well as surrounding critical organs as CTV (i.e., CTV boundaries correspond to organ boundaries in many regions, therefore, can be more reliably segmented), CTV in BT includes only the primary tumor and its potential microscopic extensions that are not identifiable in CT. Such difference in CTV led to higher DSC in their approach than our method. Automated CTV (used in BT) segmentation in MR images is challenging because, unlike CT, MR image intensity is not standardized and therefore is more variable between patients and even within the same patient. Moreover, CT does not show much soft tissue image contrast compared to MRI. Therefore, physicians may have much less flexibility in identifying the CTV boundary on CT, whereas in MR they may interpret much more visible soft‐tissue image contrast differently, leading to larger variability in manual contouring. Therefore, automatic segmentation often performs poorly with MRI than CT. For instance, automatic CTV contouring in prostate cancer, using CT images, yielded an average DSC of 0.87,^14^ whereas MR‐based method^21^ achieved a DSC of 0.83, likely for the same reason. Also, prior studies show that DL‐based OAR segmentation often performs better on CT than MRI.^28^, ^29^, ^30^ Although the comparison is indirect due to different datasets and methods used in these studies, observing the same trend in segmenting the same organs using CT and MR images supports that automated segmentation with MRI may be more challenging than that with CT. However, less variability in CT‐based segmentation does not necessarily imply more accurate CTV segmentation relative to MRI. More consistent and well‐curated training data using MRI will eventually lead to a better segmentation performance of DL‐based CTV segmentation. Wang et al.^39^ demonstrated that CT scan cannot clearly distinguish between structures of residual GTV, cervix, uterus, and vagina. Also, CT provides a poor definition of parametrial tumor infiltration.^40^ Therefore, automated CTV segmentation from MRI adds values to RT planning for cervical cancer treatment as MR is seen as the gold standard for defining the extent of tumor involvement.^2^, ^3^ Furthermore, it has been shown that for the overall staging of cervical carcinoma, MRI is more accurate than CT.^41^ Recently Yufeng et al. introduced an automatic segmentation of HR‐CTV for tandem‐and‐ovoids BT using a dual‐path 3‐D asymmetric CNN architecture with two encoding paths that was built to extract CT and MR image features.^25^ The authors used a dataset of preimplant T2W MR and postimplant CT images of 65 patients, divided into 48, 8, and 9 patients for train, evaluation, and test, respectively, to train and evaluate the CNN. The suggested methodology reported DSC of 0.65 ± 0.03, 0.79 ± 0.02, and 0.75 ± 0.04 for small tumor group (< 20 cm^3^), medium tumor group (20–40 cm^3^), and large tumor group (>40 cm^3^), respectively.

The volume of CTV ranges from 3.93 to 394.70 cm^3^ with an average of 54.69 ± 58.43 cm^3^ (mean ± SD) in our dataset. CTV volume in the test set varies from 10.63 to 123.97 cm^3^ with a mean of 47.45 ± 27.95 cm^3^. We calculated the Pearson correlation coefficient to investigate the existence of any linear relationship between CTV size and segmentation accuracy. The correlation coefficient was estimated as 0.0692, which demonstrates our proposed method, is capable of accurately segmenting CTV regardless of its size.

As the original 3‐D MRI volume is too large to fit in a GPU (unless it is very high‐end with very large GPU memory), we have to decrease the input volume size to the network. Given that the input volume size has to be the same for each network, we determined the fixed ROI size and image resolution by analyzing all of our patient data for the comparison study with different networks. For 3‐D CNN approaches (not 3‐D B2 CNN approaches), we used a large enough ROI considering the largest CTV observed in our data with added margin in order not to miss the CTV. 3‐D B2‐based networks, on the other hands, facilitate using a patient‐specific ROI as the size of the extracted block can be smaller than the CTV.

As we employed a cascaded strategy, the CTV segmentation accuracy relies on the localization validity. The proposed two‐step approach ensures that CTV is captured within ROI where ROI size was determined considering the size of the CTV and added sufficient margin not to miss the CTV. Furthermore, to address the fact that the CTV center estimated from the bladder location may not correspond to the CTV center, the CTV span detection network was trained with augmented training images using random translation, enabling robust detection performance for off‐centered ROI image. Although in all our test cases, we did not observe a noticeable failure in CTV localization, there is a possibility of such failure, in which case, the final segmentation may be suboptimal and need manual correction.

Recently, Mask R‐CNN has been introduced that can perform simultaneous ROI detection and segmentation within ROI.^42^ However, the major limitation of the Mask R‐CNN is that it requires large memory usage with often slow detection speed as each region proposal requires a full pass of convolutional networks.^43^ Some studies showed that Mask R‐CNN did not perform desirably for medical image segmentation.^44^ Vuola et al.^45^ demonstrated that the standard 2‐D U‐Net outperforms Mask R‐CNN for nuclei segmentation from a wide variety of different nucleus images, including both fluorescence and histology images of varying quality. Wang et al.^46^ demonstrated that a 2.5‐D‐based method outperforms Mask R‐CNN for liver tumor segmentation significantly. Several studies also showed that Mask R‐CNN did not perform well even for the detection task. Hussain et al.^47^ employed Mask R‐CNN to localize the kidney on CT images. Even for kidney detection, Mask R‐CNN failed to produce a proper bounding box.

Separating the detection (through coarse segmentation) and fine segmentation on the other hand has multiple advantages: (1) As mentioned before, highly modular and lighter network can be used for each step, alleviating the training burden, requiring less training data, and potentially increasing the robustness of the trained model performance. (2) As the pipeline is modular, an organ‐specific fine segmentation network with different network structure optimized to produce the best segmentation for each organ can be employed to yield overall improved segmentation performance. This flexibility enables an easier update of each organ‐specific fine segmentation network as we further develop, which is challenging if detection and segmentation models are tied and co‐trained. (3) The proposed CTV segmentation will be seamlessly combined with OAR segmentation without retraining the OAR segmentation network as the proposed method use the bladder segmentation as to guide the CTV detection process. These advantages in both implementation and update, and also performance, justify our choice of a modular cascaded coarse‐to‐fine model rather than using a combined model like Mask R‐CNN.

All the images used in this study were obtained using the same MRI scanner at a single institution, which may introduce bias into the results. It is of our interest to assess the performance of our method in multicenter trials using MR images taken with different scanners.

## 5 CONCLUSIONS

In this paper, we proposed a new fully automated DL‐based method to segment CTV from female pelvic MR images. CTV segmentation from MRI is a challenging problem as CTV encompasses microscopic cells, which are not clearly visible on MRI. Our model offers fast, accurate, and robust automatic segmentation of CTV that contributes toward cervical cancer RT planning. We used anatomy guidance in our pipeline to assist CTV localization, which significantly improved the segmentation. Experimental results confirm its superior performance compared to other state‐of‐the‐art methods. We used MCDO to produce an uncertainty map along with the segmentation, offering additional information for physicians about the quality of the segmentation and helping them to efficiently review and revise the segmentation, especially for the areas with large uncertainty.

## AUTHOR CONTRIBUTION

All the authors significantly contributed to the design of the work, image data acquisition and interpretation as well as the manuscript writing and final approval of the version to be published. Fatemeh Zabihollahy and Junghoon Lee curated image data, developed and trained the proposed deep learning model, and performed evaluation. Akila N. Viswanathan provided the expert's manual segmentations and assessed the automatic segmentation quality. Ehud J. Schmidt optimized the MR imaging sequences and performed patients' MRI scans.

## CONFLICT OF INTEREST

The authors have no conflicts of interest to disclose.
